# Genetic Influences on Cognition in Idiopathic Parkinson's Disease

**DOI:** 10.1155/2018/5603571

**Published:** 2018-08-01

**Authors:** Hanan Amer, Hatem Shehata, Laila Ahmed Rashed, Hanan Helmy, Shaimaa El-Jaafary, Asmaa Sabbah, Wael Ibrahim

**Affiliations:** ^1^Professor of Neurology, Cairo University, Egypt; ^2^Professor of Medical Biochemistry, Cairo University, Egypt; ^3^Associate Professor of Neurology, Cairo University, Egypt; ^4^Lecturer of Neurology, Cairo University, Egypt; ^5^Assistant Lecturer of Neurology, Cairo University, Egypt

## 1. Introduction

Cognitive impairment in Parkinson's disease was clearly reported in the medical literature** [1, 2].**

This impairment is common to some degree even in nondemented Parkinson's disease patients (PD-ND) and eventually progresses to dementia in 24 to 31% of patients** [3].** The cognitive changes in PD are characterized by a frontal-subcortical impairment with decreased attention and executive function leading to progressive impairment in prefrontal tasks, visuospatial skills, and memory. Still, 20 to 25% of PD-ND patients may exhibit a pattern of cortical impairment with memory tasks and confrontation naming defects, and cognitive findings associated with cortical pathology, such as language errors, develop in many patients with PD with dementia (PDD)** [4].**

The identification of the biomarkers for cognitive impairment in patients with PD will allow better assessment of the patients' prognosis. Some genes, such as apolipoprotein E (ApoE) and microtubule-associated protein tau (MAPT), are of particular interest because of their known association with dementia in other neurodegenerative diseases, such as Alzheimer's disease (AD) and atypical parkinsonian syndromes, including progressive supranuclear palsy and corticobasal degeneration** (Morley et al., 2012) [5].**

The aim of this study is to determine the role of genetic factors associated with cognitive decline in Parkinson's disease (PD). We examined whether variations in apolipoprotein E (ApoE) and microtubule-associated protein tau (MAPT) genotypes are associated with cognitive decline in PD.

## 2. Subjects and Methods

The current study is a pilot study. In this pilot study, we applied a prospective design, to evaluate cognitive changes between groups defined by genotype differences.

### 2.1. Subjects

This study included 50 patients recruited from neurology outpatient clinic and Neurology Department in Kasr AL-Ainy Hospital, Cairo, Egypt, who fulfilled the criteria for diagnosis of idiopathic Parkinson's disease based on British Brain Bank criteria** [6].**

Exclusion criteria: patients with secondary parkinsonism (drug-induced, posttraumatic, or postinfectious) or atypical parkinsonism; patients with severe dementia MMSE <11 or with major language disturbance and severe physical, auditory, or visual impairment affecting their ability to complete testing** [7];** patients with Geriatric Depression Scale (GDS) ≥ 10; patients with evidence from the history, physical examination, or investigations for any concomitant medical or metabolic illness known to affect cognition, e.g., thyroid, parathyroid, hepatic, and renal disease; patients with marked tremor, which interferes with the imaging session and produces movement artifacts, were excluded.

After assessment of cognitive functions by Mini-Mental State Examination and Parkinson's Disease-Cognitive Rating Scale (PD-CRS), the patients were divided into 2 groups; Group I included 14 patients with IPD and no cognitive impairment; Group II included 36 patients with IPD and cognitive impairment. Cognitive impairment was considered if there was an abnormality in more than one cognitive domain, representing a decline from premorbid level.

The study was approved by Neurology Department Board in Cairo University and follows the principles outlined in the Declaration of Helsinki. Verbal informed consent was obtained from all patients prior to the commencement of the study after a structured interview clarifying the aim and steps of the study.

### 2.2. Methods

All patients in this study were submitted to the following: thorough history taking and neurological examination according to the standardized sheet of Neurology Department, Kasr Al-Ainy Hospital.

All patients were evaluated for Parkinson's disease severity while on their best state using** Unified Parkinson's Disease Rating Scale** (UPDRS) [8].

**Neuropsychological assessment scales:** MMSE** [7]**, GDS** [9],** and Parkinson's Disease-Cognitive Rating Scale (PD-CRS) [4] were applied to our patients in this study.

Genotyping: all the patients were genotyped for the Apo E and MAPT polymorphism.

Steps of gene polymorphism detection by real-time PCR: (A) DNA extraction and (B) genotyping.

Genotyping was performed using real-time polymerase chain reaction with TaqMan allelic discrimination assay (Applied Biosystems, USA). Genotyping for (rs1052553, C_7563736) MAPT allelic variant and ApoE alleles (rs7412, C_904973_10 and rs429358, C_3084793_20) single nucleotide polymorphisms was performed using real-time polymerase chain reaction.

PCR amplification protocol: a predesigned primer/probe set for the genotypes was used (Applied Biosystems, USA). Probes were synthesized with reporter dye FAM or VIC covalently linked at the 5 and a quencher dye MGB linked to the 3 end of the probe (Applied Biosystems, USA).

PCR mix: DNA amplification was carried out in a 25 *μ*l total volume containing 12.5 *μ*l Taqman master mix, 1.25 primer/probe, 1 *μ*l DNA, and 10.25 H2O.

Cycling conditions: real-time PCR was performed using Applied BioSystem step one plus Real-Time PCR System (Applied BioSystem, CA, USA) with the following conditions: after a denaturation time of 10 min at 95°C, 45 cycles at 92°C for 15s, and then 60°C for 90s for annealing and extension were carried out and fluorescence was measured at the end of every cycle and at the endpoint.

### 2.3. Statistical Methods

Data was coded and entered using the statistical package (SPSS) version 12. Data was summarized using mean and standard deviation for quantitative variables and percent for qualitative variables. Categorical variables were compared with chi square test, and continuous variables were compared with t-test (normal distribution) or Mann–Whitney tests (nonnormal distribution). A value < 0.05 was set as significant and <00.1 as very significant.

## 3. Results

The current study included 50 patients divided into 2 groups with their demographic and baseline characteristics described in Table 1.

The results showed the presence of significant differences concerning the age, scores of UPDRS, MMSE, and PDCRS between the 2 groups, representing that the group with cognitive changes included older patients with higher UPDRS score.

Patients were divided clinically into tremor predominant or rigidity predominant. In Group I, 13 patients (92.9%) were tremors predominant, while in Group II, 24 patients were tremors predominant (66.7%) (P=0.078). 

**Figure 1:**
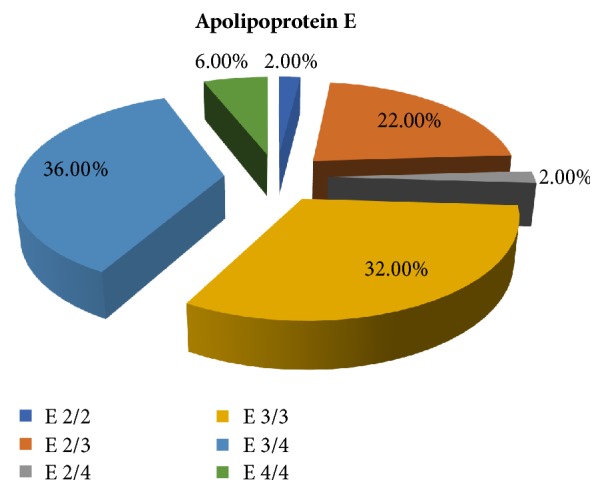
Percentage of different ApoE alleles in all patients.

*Apolipoprotein E Polymorphism*. All PD patients were genotyped for ApoE polymorphism alleles *ε*2/2, *ε*2/3, *ε*2/4, *ε*3/3, *ε*3/4, and *ε*4/4, as illustrated in Figure 1. Most of the patients were *ε*3/3 and *ε*3/4 genotype representing 23% and 36%, respectively.

In Group I, the *ε*4 carrier (n=7) represents 50% which is equal to *ε*4 noncarrier (n=7) which represents 50%, while in Group II, the *ε*4 carrier (n=15) represents smaller proportion, 41.7%, to *ε*4 noncarrier (n=21) which represents 58.3%, as illustrated in Table 2. 

*MAPT Polymorphism*. All PD patients were genotyped for MAPT polymorphism haplotypes H1/H1, H1/H2, and H2/H2.

**Figure 2:**
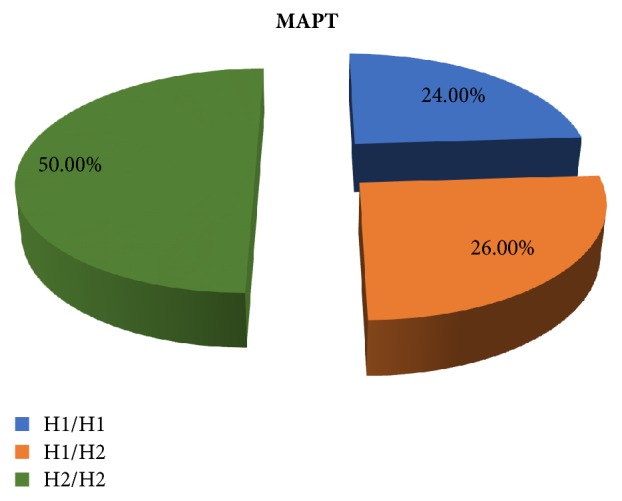
Percentage of different MAPT haplotypes in all patients.

Fifty percent of the patients were H2/H2 genotype and 24% were H1/H1 genotype, as illustrated in Figure 2.

In Group I almost all of the patients were homozygous either H1/H1 (50%) or H2/H2 (42%) but in Group II, 33.3% were heterozygous H1/H2, while H2/H2 homozygous was 52.8% (P-value=0.02).  Table 3 shows the different MAPT haplotypes in both groups.

### 3.1. MMSE and Genotypes in Groups I and II Patients at 0 and 3 Months

*Group I at 0 months:* the mean MMSE was 29 ± 0.577 in ApoE *ε*4 carrier which is lower than in *ε*4 noncarrier 29.57 ± 0.543. The mean MMSE was 29.42 ± 0.786 in MAPT H1/H1 haplotype which is higher than the other haplotypes 29.14 ± 0.377.* Group I at 3 months:* the mean MMSE decreased by 1.86 points in ApoE *ε*4 carrier and by 0 points in *ε*4 noncarrier (p-value=0.004). The mean MMSE decreased by 1.14 points in MAPT H1/H1 haplotype and by 0.75 points in other haplotypes.

**Figure 3:**
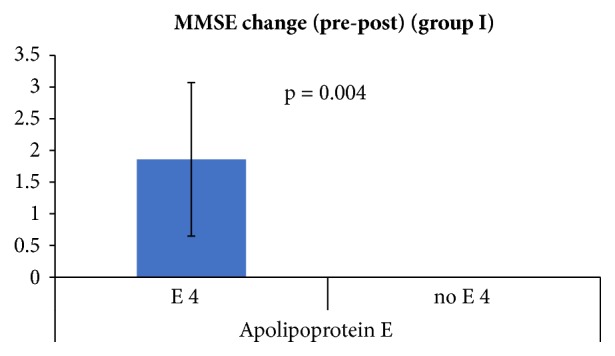
Group I mean MMSE change in ApoE *ε*4 carrier/noncarrier.

**Figure 4:**
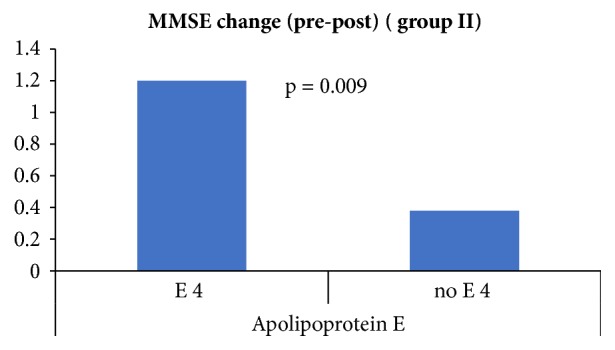
Group II mean MMSE change in ApoE *ε*4 carrier/noncarrier.

*Group II at 0 months:* the mean MMSE was 23.80 in ApoE *ε*4 carrier which is nearly the same as in *ε*4 noncarrier 23.80. The mean MMSE was 24.40 in MAPT H1/H1 haplotype which is higher than in other haplotypes 23.70.* Group II, at 3 months:* the mean in MMSE decreased by 1.20 points in ApoE *ε*4 carrier and by 0.38 points in *ε*4 noncarrier. The mean MMSE decreased by 1.40 points in MAPT H1/H1 haplotype and by 0.61 points in other haplotypes, as illustrated in Figures 3 and 4.

### 3.2. PDCRS and Genotypes in Groups I and II Patients at 0 and 3 Months

Group I: in ApoE *ε*4 carrier mean PDCRS total score was 78.85 at 0 months, which is slightly better than in *ε*4 noncarrier 77.57 (p value =0.89). MAPT H1/H1 haplotype patients had a mean PDCRS total score of 83.28 at 0 months which was better than other haplotypes 73.14 (P value = 0.26).

As regards the subscales of PDCRS, (i) the mean scores in PDCRS frontosubcortical subscale changed by 6 points in ApoE *ε*4 carrier and by 0.86 points in *ε*4 noncarrier. (ii) The mean scores of the PDCRS posterior-cortical subscale changed by 1.43 points in *ε*4 carrier and by 0 points in *ε*4 noncarrier. (iii) The mean PDCRS total scores changed by 7.43 points in *ε*4 carrier and by 0.86 points in *ε*4 noncarrier.

**Figure 5:**
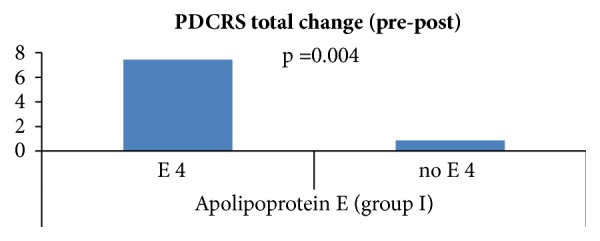
Group I mean PDCRS change in ApoE *ε*4 carrier/noncarrier group.

A statistical significance was found between changes in PDCRS frontosubcortical and ApoE *ε*4 carrier/noncarrier (p-value=0.004), and between change in PDCRS total and ApoE *ε*4 carrier/noncarrier (p-value=0.004) with PDCRS score worse with ApoE *ε*4 carriers, Figure 5. However, no statistical significance was found between change in PDCRS posterior-cortical and ApoE *ε*4 carrier/noncarrier.

Group II: the mean PDCRS total score at 0 months (54.66) is better than in *ε*4 noncarrier (43.71), p value = 0.1. The mean PDCRS total score at 0 months in MAPT H1/H1 (61.20) is better than in other haplotypes (46.19), p-value = 0.11.

**Figure 6:**
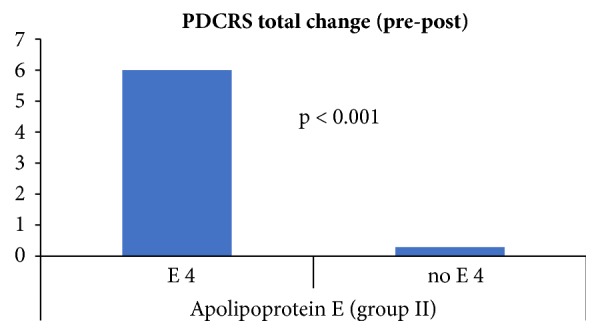
Group II mean PDCRS change in ApoE *ε*4 carrier/noncarrier.

Regarding the PDCRS subscales, (i) the mean PDCRS frontosubcortical scores changed by 3.67 points in ApoE *ε*4 carrier and by 0.14 points in *ε*4 noncarrier. (ii) The mean PDCRS posterior-cortical scores changed by 2.33 points in *ε*4 carrier and by 0.14 points in *ε*4 noncarrier. (iii) The mean PDCRS total scores changed by 6 points in *ε*4 carrier and by 0.29 points in *ε*4 noncarrier, as illustrated in Figure 6.

A high statistical significance was found between changes in PDCRS frontosubcortical, PDCRS posterior-cortical, PDCRS total, and ApoE *ε*4 carrier/noncarrier (p-value < 0.001) with PDCRS score worse with ApoE *ε*4 carriers.

The mean in PDCRS frontosubcortical scores changed by 1.80 points in MAPT H1/H1 haplotype and by 1.58 points other haplotypes. The mean PDCRS posterior-cortical scores changed by 1 point in MAPT H1/H1 haplotype and by 1.06 points in other haplotypes. The mean PDCRS total changed by 2.80 points in MAPT H1/H1 haplotype and by 2.65 points in other haplotypes.

No statistical significance was found between change in PDCRS frontosubcortical, PDCRS posterior-cortical, PDCRS total, and MAPT H1/H1 haplotype (p-value=1, p-value=0.69, and p-value=0.723, respectively).

### 3.3. Comparison of Apolipoprotein *ε*4 Carrier between Groups I and II Patients in Different Rating Scales

**Figure 7:**
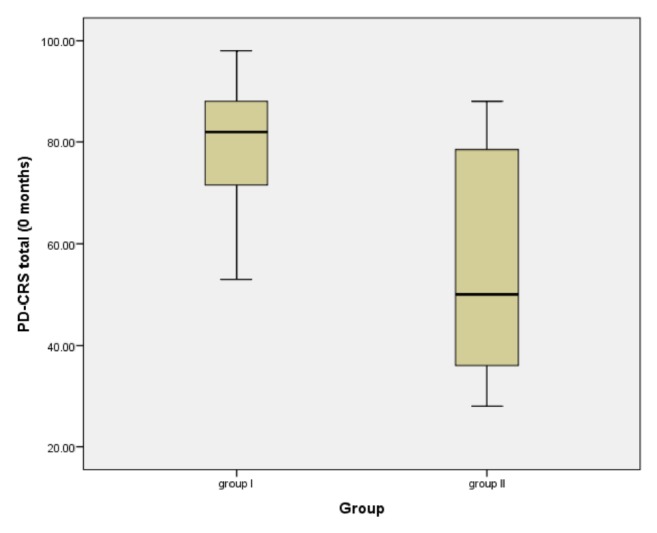
Mean score of PDCRS for ApoE *ε*4 carriers between Group I and Group II.

The cognitive performance was better in ApoE *ε*4 carriers in Group I than in Group II as evident by the mean MMSE score and mean PDCRS (total) score at 0 months, as illustrated in Figure 7. A statistical significance was detected between both groups (p-value=<0.001 and 0.017, respectively). The motor functions were worse in ApoE *ε*4 carriers in Group II than in Group I as evident by the mean UPDRS score; a statistical significance was detected between both groups (p-value=0.041).

### 3.4. Comparison of MAPT H1/H1 Haplotype between Groups I and II Patients in Different Rating Scales

**Figure 8:**
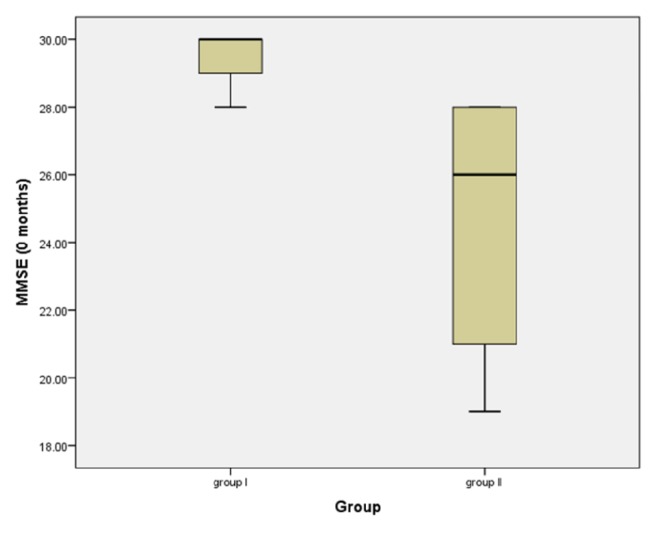
Mean score of MMSE for MAPT H1/H1 haplotype patients between Group I and Group II.

The cognitive performance was better in MAPT H1/H1 haplotype in Group I than in Group II as evident by the mean MMSE score (Figure 8) and mean UPDRS (total) score (at 0 months); a statistical significance was detected between both groups regarding MMSE (p-value=0.053) but not with PDCRS.

The motor functions were worse in MAPT H1/H1 haplotype in Group II than in Group I as evident by the mean UPDRS score, but no statistical significance was detected between both groups.

### 3.5. Comparison of Combined Genotypes with Different Rating Scales

Out of the 50 patients included in the study, 20 PD patients had either single ApoE *ε*4 carrier or MAPT H1/H1 and 7 patients had both ApoE *ε*4 carrier and MAPT H1/H1.

The mean MMSE changed by 0.90 ± 0.85 points in ApoE *ε*4 carrier or MAPT H1/H1 and by 2 ± 1.29 points in ApoE *ε*4 carrier and MAPT H1/H1 (p=0.48).

The change in mean UPDRS I and mean UPDRS total changes in “ApoE *ε*4 carrier or MAPT H1/H1” and in “ApoE *ε*4 carrier and MAPT H1/H1” were not statistically significant as shown in Table 4.

The mean change in PDCRS frontosubcotical in “ApoE *ε*4 carrier/or MAPT H1/H1” group was 2.45 ± 3.41 points, while in the “ApoE *ε*4 carrier and MAPT H1/H1” it was 6.14 ± 3.13 points (p=0.019).

The mean change in PDCRS total in “ApoE *ε*4 carrier/or MAPT H1/H1” group was 4.05 ± 4.74 points, while in the “ApoE *ε*4 carrier and MAPT H1/H1” group, it was 7.8 ± 3.72 (p= 0.031).

The change in PCDRS posterocortical was not statistically significant as illustrated in Table 4. There was no statistical significant correlation between duration of illness and both UPDRS and PDCRS, as illustrated in Table 5.

## 4. Discussion

Cognitive impairment is a common and functionally significant problem in PD, with a cumulative prevalence of dementia as high as 75%–90% [10].

The MMSE score in our study patients ranged from 12 to 30. In group I, the mean MMSE decreased by 0.93 points in 3 months, while in Group II the mean MMSE decreased by 0.73 points in 3 months (a nonsignificant difference). Cognitive impairment in PD has been clearly reported in the medical literature. The exact pattern of this impairment and its frequency are still a subject of considerable controversy. In our study, the mean PDCRS frontosubcortical change was 2.12 points compared to the mean PDCRS posterior-cortical change which was 0.96 points in all patients after 3 months of follow-up. The frontosucortical patterns of cognitive impairment in PD patients were also recognized by** Muslimovic et al. (2005) [2]**in which there are decreased attention and executive function together with the impairment in visuospatial skills and memory.

In the current study, a negative correlation is observed between the age of onset of PD symptoms and PDCRS in group I (r= -0.368, p= 0.195) and Group II (r= -0.188, p= 0.273).** Dubois et al. (1991) [11]**did not find significant association between increasing age and cognitive impairment in patients with PD. They attributed this to the effect of the degenerative process in PD which may be more prominent than the effect of aging on cognitive functions.** Caviness et al. (2007) [12] and Verbaan et al. (2007) [13] highlighted** a trend for a significant role of disease duration in developing cognitive impairment in patients with PD.

UPDRS has been the most widely used scale to measure impairment and disability in PD** [14].**

The UPDRS (total) score in patients of this study ranged from 10 to 72. In Group I, the mean UPDRS increased by 5.14 points after 3 months of follow-up. In Group II, the mean UPDRS increased by 3.98 points after 3 months of follow-up. A statistically significant difference was observed between Groups I and II as regards the UPDRS at baseline assessment and after 3 months of follow-up (p-value; 0.018 and 0.025, respectively).

In a study performed by** Campos et al., 2015 **for factors predicting dementia, they found that motor performance as assessed by the (UPDRS-Part III) was a strong predictor, whereas, neither age nor Hoehn and Yahr scale (H&Y) scoring was predictors** [15]**. On the contrary,** Stavitsky et al. (2011) **found that UPDRS (total) and sleep did not predict a future memory decline [16].

In this study, the decline in cognition after 3 months in Group I patients was 4.14 points (in PDCRS) in relation to worsening of motor symptoms by 1.85 points (in UPDRS III) and in Group II patients where decline in cognition was 2.67 points (in PDCRS) in relation to worsening of motor symptoms by 0.75 points (in UPDRS III); an inverse relationship is observed between UPDRS III (motor) and PDCRS (total), as the UPDRS III score increases (motor symptoms worsen) the PDCRS score decreases (cognitive symptoms worsen). A statistical significant correlation between UPDRS III and PDCRS (total) (p-value=0.003) in all PD patients was in agreement with studies by** Muslimovic et al., 2005 [3], Verbaan et al., 2007 [13], Mamikonyan et al., 2009 [17], **and** Aarsland et al., 2010 **[18] who reported a significant association between the severity of motor symptoms in patients with PD and the development of cognitive dysfunction. Such association was explained by the presence of frontostriatal circuits dysfunction in patients with PD which in turn is responsible for producing both the motor symptoms and cognitive impairment [19]. On the other hand,** Cooper et al. 1991 [20]**and** Abo-El-Naga 2006 [21]** failed to establish a relationship between motor function and cognitive impairment in patients with PD. The investigators defended their findings by concluding that cognitive dysfunction in early PD patients reflected neuropathological changes that are distinct from those responsible for the motor disorder.

There is a continuous search for biological markers of cognitive dysfunction in Parkinson's disease, which encouraged the investigation of the genetic effect; some of the studied genes were previously reported to be associated with cognitive impairment in PD, through affecting the dopaminergic systems. Other genes, ApoE and MAPT, are of particular interest because of their known association with dementia in other neurodegenerative diseases, such as Alzheimer's disease (AD) and atypical parkinsonian syndromes, including progressive supranuclear palsy and corticobasal degeneration** [5].**

Relatively few studies examined the* MAPT* H1 haplotype as a risk factor for cognitive impairment in PD. All PD patients in our study were genotyped for MAPT polymorphism. In Group I, the H1/H1 haplotype (n=7) represents 50%, while in Group II the H1/H1 haplotype (n=5) represents a much smaller proportion 13.9% to other haplotypes (n=31) which represent 86.1%. A statistical significance was found between different haplotypes (P-value=0.020) among Groups I and II patients. Contrary to our findings,** Ezquerra et al. [22]** found no difference in H1 frequency between PD patients with and without dementia. The results of the current study showed a faster rate of decline in MMSE scores in individuals with the MAPT H1 variant. Although no statistical significance was found between change in MMSE and MAPT haplotypes, yet, the mean MMSE change was higher in patients with MAPT H1/H1 haplotype compared to those with other haplotypes, being 1.14 (group I) and 1.40 (Group II) points in the former and 0.75 (group I) and 0.61 (Group II) points in the later. A longer follow-up with such decline rates might show a significant decline in MMSE score in MAPT H1/H1.

We also concluded that the changes in PDCRS frontosubcortical, PDCRS posterior-cortical, PDCRS total were worse in the MAPT H1/H1 haplotype compared to other haplotypes as measured by PDCRS without statistical significance. These results are similar to some extent to a study conducted by** Williams-Gray et al., 2009 [23]**, which included a cohort of 122 PD patients who were followed up for 5 years; they found that MAPT-H1 haplotype was associated with a more rapid decline in Mini-Mental State Examination score and was a significant risk factor for conversion to dementia. On the contrary,** Morley et al. (2012) [5] did** not find a significant association between MAPT haplotype and rates of cognitive decline.

In this study, the *ε*4 carrier (n=7) represents 50% which is equal to *ε*4 noncarrier (n=7) which represent 50% in PD patients without cognitive impairment, while, in Group II, the *ε*4 carrier (n=15) represents smaller proportion, 41.7%, to *ε*4 noncarrier (n=21) which represents 58.3%.

We found that worsening of cognition was more with ApoE *ε*4 carrier than with *ε*4 noncarrier where a statistical significance was found between change in PDCRS and ApoE *ε*4 carrier/noncarrier as the mean PDCRS total change by 7.43 (in Group I) and 6 (in Group II) points in *ε*4 carrier and by 0.86 (in Group I) and 0.29 (in Group II) points in *ε*4 noncarrier.** Harhangi et al., 2000 [24]; Parsian et al., 2002 [25], and Pankratz et al., 2006 [26]**came with similar results. In contrast, other studies as** Williams-Gray et al., 2009 [23]; Ezquerra et al., 2008 [22]; **and** Ryu and kwon, 2010 [27]** failed to find a correlation.

The observed association between ApoE and cognitive decline may not be specific to PD and it could be observed in otherwise healthy older individuals. This was previously discussed by** Morley et al., 2012** [5] who could not establish a confirmed association.

In conclusion, patients in Group I with no cognitive dysfunction showed statistically significant worsening in PDCRS frontosubcortical but not in PDCRS posterior-cortical, while patients in Group II with cognitive dysfunction showed significant worsening in PDCRS frontosubcortical and PDCRS posterior-cortical, and this result of early involvement of the frontosubcortical tests rather than posterior-cortical which is affected later in the disease with development of dementia. In this study, 27 PD patients have ApoE *ε*4 carrier and/or MAPT H1/H1. PD patients with ApoE *ε*4 carrier or MAPT H1/H1 represent larger proportion (75%) compared to PD patients with both ApoE *ε*4 carrier and MAPT H1/H1 (n=7) represents 25%. A statistical significance was found between change in MMSE (p-value < 0.048) and ApoE *ε*4 carrier and MAPT H1/H1. A statistical significance was found between change in PDCRS frontosubcortical (p-value=< 0.019), PDCRS total (p-value=< 0.031), and carriers of both ApoE *ε*4 and MAPT H1/H1 with scores of cognitive functions being worse with carriers of both ApoE *ε*4 and MAPT H1/H1 more than carriers of either ApoE *ε*4 or MAPT H1/H1.

## Figures and Tables

**Table 1:** Demographic data and baseline characteristics of the study population.

	Group I		Group II		P value
	*No./range*	*Mean ±SD*	*No./ range*	*Mean ±SD*	
Age	(50-77)	57.79 ± 7.79	(51-85)	62±44	0.019*∗*
Gender	M 71.4%, F 28.6%		M 66.7%, F 33.3%		1
Age of onset	(45-67)	54.50 ± 8.93	(46-75)	58.33 ± 6.52	0.719
Duration of illness	(0.5-9)	3.29 ± 2.31	(0.5-20)	4.11 ± 3.99	0.066
UPDRS_0	14	26.93 ± 13.64	36	38.83 ± 15.86	0.018*∗*
GDS _0	14	5.14 ± 2.6	36	5.75 ± 283	0.479
MMSE _0	14	29.29 ± 0.61	36	23.81 ± 4.39	0.001*∗*
PDCRS_ 0	14	78.21 ± 16.63	36	48.28 ± 19.72	0.001*∗*

*∗* P ≤ 0.05 significant. UPDRS: Unified Parkinson's Disease Rating Scale, GDS: Geriatric Depression Scale, MMSE: Mini-Mental State Examination, and PDCRS: Parkinson's Disease Cognitive Rating Scale.

**Table 2:** Number and percentage of different ApoE genotypes in Group I and Group II patients.

Genotypes		group I		group II		P value
		No	%	No	%	
ɛApo E	ɛ 2/2	1	7.1	0	0	0.149
	ɛ 2/3	4	28.6	7	19.4	
	ɛ 2/4	0	0	1	2.8	
	ɛ 3/3	2	14.3	14	38.9	
	ɛ 3/4	5	35.7	13	36.1	
	ɛ 4/4	2	14.3	1	2.8	

**Table 3:** Number and percentage of different MAPT haplotypes in Group I and Group II patients.

Genotype		Group I		Group II		P value
		No	%	No	%	
MAPT	H1/H1	7	50	5	13.9	0.020*∗*
	H1/H2	1	7.1	12	33.3	
	H2/H2	6	42.9	19	52.8	

*∗* p-value < 0.05 (significant).

**Table 4:** The mean change in different assessment scales (between 0 and 3 months) of PD patients with different combination of genotypes in Group I and Group II patients.

	ApoE *ε*4 carrier/or		ApoE *ε*4 carrier and		P value
	MAPT H1/H1		MAPT H1/H1		
	Mean	SD	Mean	SD	
MMSE	0.90	0.85	2.00	1.29	0.048*∗*
UPDRSI	-0.95	1.00	-0.71	1.11	0.808
UPDRS total	-4.75	4.96	-6.57	5.0	0.288
PDCRS fronto- subcortical	2.45	3.41	6.14	3.13	0.019*∗*
PDCRS postero-cortical	1.60	2.19	1.71	1.38	0.341
PDCRS total	4.05	4.74	7.86	3.72	0.031*∗*

*∗*  p-value < 0.05 (significant).

**Table 5:** Correlations between the duration of illness and rating scales in Group I and Group II patients.

	Group I			Group II		
	No	Correlation coefficient	P value	No	Correlation coefficient	P value
PDCRS	14	-0.175	0.550	36	-0.032	0.852
UPDRS	14	0.208	0.476	36	0.055	0.749

## Data Availability

The data used to support the findings of this study are available from the corresponding author upon request.
